# United States Department of Agriculture

**Published:** 1897-10

**Authors:** A. J. Wedderburn, James Wilson

**Affiliations:** Special Agent; Washington, D. C.; Secretary; Washington, D. C.


UNITED STATES DEPARTMENT OF AGRICULTURE.

DIVISION OF CHEMISTRY.

Washington, D. C., September 17th, 1897.

Dear Sir:

Under authority of Congress, the Department of Agriculture is investigating the extent and character of food and drug adulterations, and is desirous of securing all the information possible on the subject. Having been appointed special agent to inquire into and report upon this matter, the undersigned writes to request that you kindly furnish the Department (under the inclosed franks) all the information you have in regard to adulterations, together with any suggestions as to the best remedy for the evil.

(1) Do you know of any new adulterant? If yes, state what, and how used.

(2) Would a national food and drug law assist in preventing adulteration?

(3) Would uniform food, drug, and pharmaceutical laws tend to promote efficiency and purity?

(4) Please suggest what would best promote the interests of consumers and legitimate manufacturers and dealers.

(5) What is your opinion as to the extent of damage done legitimate business by imitation of brands, packages, etc.?

(6) To what extent do sophistication, misbranding, and injurious adulteration exist?

(7) Have State laws aided in preventing adulteration? To what extent?

(8) Would a national law assist State officials in properly executing the local laws?

(9) Have adulteration, sophistication, and misbranding increased or decreased?

Prompt replies to the above, together with any other information or suggestions, will be highly appreciated.

Yours respectfully,

A. J. WEDDERBURN,

*Special Agent.*

Approved:

JAMES WILSON,

*Secretary.*

Inclosure.

Please write answers on reverse side of this sheet and return to the special agent.

